# MAPK1 of *Leishmania donovani* interacts and phosphorylates HSP70 and HSP90 subunits of foldosome complex

**DOI:** 10.1038/s41598-017-09725-w

**Published:** 2017-08-31

**Authors:** Pavneet Kaur, Mansi Garg, Antje Hombach-Barrigah, Joachim Clos, Neena Goyal

**Affiliations:** 1 Division of Biochemistry, CSIR-Central Drug Research Institute, Sector 10, Jankipuram Extension, Sitapur Road, Lucknow, 226031 Uttar Pradesh India; 2 Bernhard Nocht Institute for Tropical Medicine, D-20359 Hamburg, Germany

## Abstract

MAP kinases (MAPK) are the most downstream kinases in signal transduction cascades and regulate critical cellular activities such as cell proliferation, differentiation, mortality, stress response, and apoptosis. The *Leishmania donovani* MAPK1 (LdMAPK1) is involved in parasite viability and drug resistance, but its substrates have not been identified yet. Aiming to identify the possible targets(s) of LdMAPK1, we sought to isolate interacting partners by co-immunoprecipitation, gel electrophoresis and mass spectrometry. Out of fifteen analyzed protein bands, four were identified as subunits of the HSP90 foldosome complex, namely HSP 90, HSP70, STI and SGT. Western blot analysis not only confirmed that LdMAPK1 interacts with HSP70 and HSP90 but also demonstrated that MAPK1 abundance modulates their expression. The interaction is sensitive to treatment with AMTZD, a competitive ERK inhibitor. MAPK1 also displayed kinase activity with HSP90 or HSP70 as substrates. By phosphorylating HSPs in the foldosome complex, MAPK1 may regulate the stability and activity of the foldosome which in turn plays a pivotal role in the parasitic life cycle of *L. donovani*. Our study therefore implicates LdMAPK1 in the post-translational modification and possibly the regulation of heat shock proteins. Conversely, HSP90 and HSP70 are identified as the first substrates of LdMAPK1.

## Introduction

Protozoan parasites of the genus *Leishmania* are the causative agents of a range of human disease, from self-curing, ulcerative skin lesions (Cutaneous Leishmaniasis, CL), and destructive mucosal inflammation (Muco-cutaneous Leishmaniasis, MCL) up to the fatal hepato-splenomegaly (Visceral Leishmaniasis, VL)^1^. According to WHO reports, more than 1.3 million new cases and 20,000 to 30,000 deaths per year are reported globally, with 310 million people at risk of an infection. Since vaccines are still under development, the control of the disease relies on chemotherapy^2^ and control of the arthropod vectors, sand flies of the genera *Phlebotomus* and *Lutzomyia*. For decades, sodium stibogluconate and meglumine antimoniate have been the first line therapy against this parasitic infection. Second line treatments include the use of amphotericin B, pentamidine, paramomycin and the oral drug miltefosine^3^. Unfortunately, the emergence of resistance to the first line drugs and the toxicity, high cost and developing resistance to the second line drugs raise concerns over the available treatment options for leishmaniasis^4^. Another challenge is the coinfection with leishmaniasis and HIV, which defies existing treatments^5^. The present day need is to battle the spread of drug resistance and to combine efforts to formulate new drugs and drug combinations.

*Leishmania* is a dimorphic protozoan parasite that lives in two forms in the sand fly vectors and the mammalian hosts. In the sandflies, the parasites reside as flagellated promastigotes in the gut lumen. In mammalian hosts, the parasites multiply as aflagellated amastigotes within macrophages^6^. The conversion from the promastigote (procyclic, metacyclic) to the amastigote stage and vice versa is pivotal for parasite survival and pathogenesis, and is linked to the parasite’s response to the environmental changes during their transmission from poikilothermic, hematophagous insects to homeothermic mammals and vice versa. This differentiation process involves not only a morphological change but also the retooling of metabolic processes which are reflected in changes to the proteome^7^. During the differentiation of *Leishmania* parasites from one stage to another, proteins undergo post translational modifications (PTMs), such as methylation, glycosylation, fucosylation, acetylation, and phosphorylation. Protein phosphorylation is one of the most studied modifications in eukaryotes given its relevance in regulating important cellular events such as gene transcription, cell morphology, or the cell cycle^8^. Therefore, phosphoproteome analysis has been performed to identify phosphorylation events that correlate to parasite differentiation^9–11^. Phosphorylated proteins mostly include stress and heat shock protection proteins, ribosomal subunits, RNA helicases and RNA binding proteins, protein kinases, phosphatases and various metabolic and cytoskeletal proteins^9, 12^.

Out of the 179 identified protein kinases in *Leishmania*, 15 genes are established as typical MAPKs^13^. However, for only few of them, a functional role in the parasite’s life cycle could be established. The *L. mexicana* MAPK1 (LmxMPK1) was found to be essential for survival of amastigotes within the mammalian macrophages^14^. In addition, MAPK1 was also shown to play a role in antimony resistance^15^. LmxMPK3 and LmxMPK9 are involved in the length regulation of promastigote flagella^16, 17^, while LmxMPK4 is involved in stage conversion and affects virulence of *L. mexicana* ^18^. Even less is known about the MAPK-dependent pathways, their upstream activators and their downstream effectors. LmxMPK3 is phosphorylated by LmxMPKK^16^ while LmxMPK4 is known to be phosphorylated by LmxMKK5^19^. Recently, MAPK2 was shown to phosphorylate AQP1, the influx pump for trivalent antimony, thus increasing the intracellular drug accumulation and resulting in increased parasite antimony sensitivity^20^.

In the present study, we explored the possible targets(s) of LdMAPK1 and established that LdMAPK1 interacts with the subunits of the *Leishmania* HSP90 foldosome complex. The HSP70 and HSP90 subunits of this complex are the possible substrates for LdMAPK1, suggesting an important role for this kinase in parasite survival and life cycle control.

## Results

### MAPK1 interacts with the HSP90 foldosome complex

**Figure 1:**
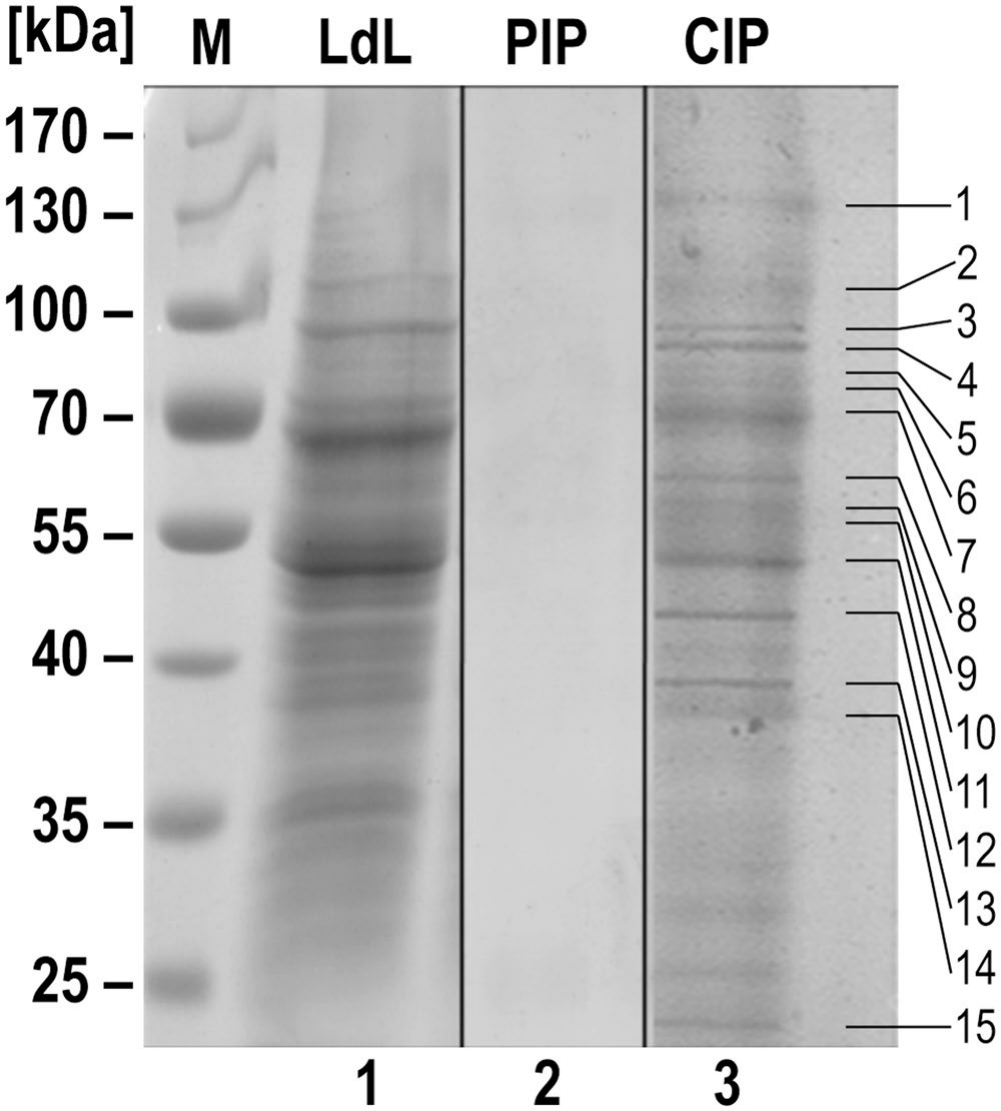
Identification of LdMAPK1 interacting partners through coimmuno-precipitation. The whole cell lysates were immune-precipitated with either pre-immune sera (PIP) or with MAPK1 polyclonal antibody (CIP). Whole cell lysate (LdL) and immunoprecipitates were separated on SDS-PAGE and stained with Colloidal Coomassie Brilliant Blue G-250 (lanes1–3). The molecular masses (kDa) are indicated on left of marker lane (M). Numbers (1–15) denote differentially expressed protein bands that have been processed for MALDI-TOF MS/MS analysis.

We investigated possible target protein(s) for LdMAPK1 using anti-LdMAPK1 antibodies in a conventional co-immunoprecipitation approach. SDS-PAGE analysis of the precipitated proteins separated several proteins ranging from 13 to 130 kDa (Fig. 1). The 15 most prominent bands were excised from the gels and identified using MALDI-MS/MS mass spectrometry (Table 1). In addition to MAPK1, several subunits of the foldosome complex, namely HSP90, HSP70, STI1 and SGT were present in the co-immunoprecipitate, indicating that MAPK1 may interact with one or more of the components of the HSP90 foldosome complex described by Buchanan^21, 22^. Other possible interacting partners were identified as elongation factor 2, full ATP synthase subunit alpha, beta tubulin and several hypothetical proteins.

**Table 1:** MAPK1-associated proteins isolated by co-immunoprecipitation were identified through MALDI-MS/MS analysis.

ID	Protein name	Accession number	MW (Dalton)	Score	No. of peptide sequences
1	Hypothetical protein	gi │308162183	121453	52*	2
2	Hypothetical protein	gi │146096880	112511	58	1
3	Elongation factor 2	gi │146103554	94942	201	10
4	HSP90	gi │321398592	81013	747	17
5	Hypothetical protein	gi │167516232	75545	67*	2
6	Hypothetical protein	gi │328871460	74904	45*	2
7	HSP70	gi │123591	71410	161	6
8	STI 1	gi │146078042	62710	286	12
9	Full ATP synthase subunit alpha	gi │399078	54683	103	2
10	Hypothetical protein	gi │146105034	58765	70*	4
11	Beta tubulin, Full elongation factor 1- alpha	gi │1296832, gi │119148	50302 48981	43, 42	4, 2
12	SGT (small glutamin-rich tetratricopeptide repeat protein)	gi │146094018	45956	342	8
13	MAPK	gi │146102593	41270	324	9
14	Unnamed protein product	gi │322501241	39332	251	6
15	Hypothetical protein	gi │261328516	13000	46*	2

*Symbol indicates that the protein identification is below significance levels.

**Figure 2:**
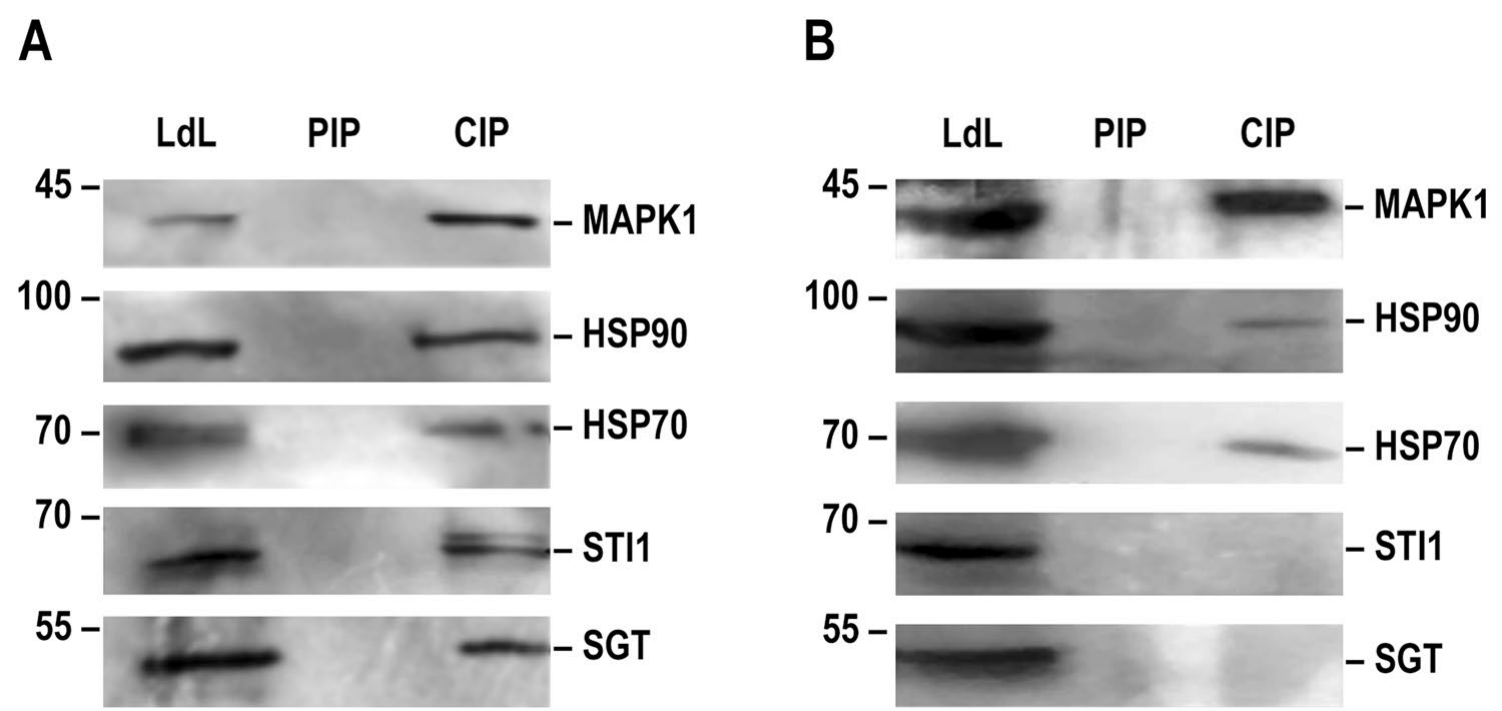
Western blot analysis of co-immunoprecipitation with anti-LdMAPK1 polyclonal antibody (**A**) before and (**B**) after treatment with geldanamycin. LdL: *L. donovani* whole cell lysate, PIP: co-immunoprecipitate with preimmune sera, CIP: co-immunoprecipitate with anti-LdMAPK1 sera. Proteins were separated on SDS-PAGE and analyzed by western blotting with anti-LdMAPK1, anti-HSP90, anti-HSP70, anti-STI1 and anti-SGT antibodies, separately.

The molecular interactions of Ld MAPK1 with chaperone and co-chaperone subunits of the foldosome complex were further confirmed by western blotting (Fig. 2A). It was confirmed that the co-immunoprecipitate of MAPK1 contained at least 4 foldosome complex subunits (HSP70, HSP90, STI1 and SGT), all of which are essential for *Leishmania* viability.

Since the HSP90 foldosome is a rather stable complex, LdMAPK1 must not necessarily bind directly to all its components. Therefore, to identify the directly interacting partner(s) of MAPK1, the cells were treated with 100 nM geldanamycin. Geldanamycin inhibits the ATPase activity of HSP90, thus destabilizing the foldosome complex. The co-immunoprecipitate of MAPK1 from geldanamycin-treated promastigotes contained only HSP70 and HSP90 (Fig. 2B). Absence of STI1 and SGT in the co-immunoprecipitate suggests that these co-chaperones are interacting indirectly with MAPK1, via HSP90 and/or HSP70.

### MAPK1 abundance affects the expression of HSP70 and HSP90

**Figure 3:**
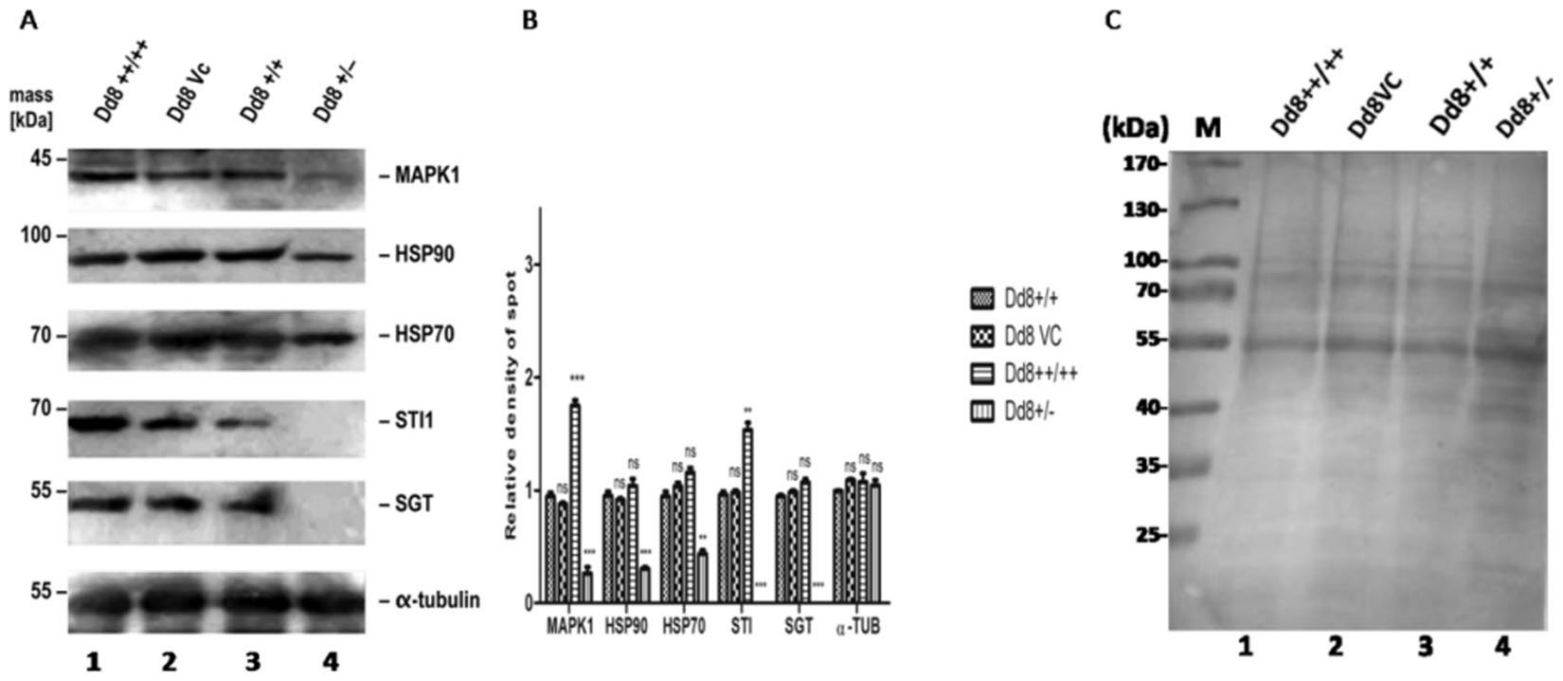
Changes in expression of chaperones (HSP90 and HSP70) and co-chaperones (STI1 and SGT) upon modulation of MAPK1 abundance. The modulation was observed in *L. donovani* promastigotes: wild type (Dd8+/+), vector control (Dd8 Vc), over-expressing transfectants (Dd8++/++), and single deletion mutants (Dd8 + /−). (**A**) Western blots showing expression of molecular chaperones in differentially expressing MAPK1 cells (lanes1-4). TUB (α-tubulin) was taken as the control. (**C**) Ponceau S staining of membrane for total protein prior to antibody incubations. The molecular masses (kDa) are indicated on the left.

It was not possible to raise viable MAPK1 null mutants of *L. donovani* (M.G. unpublished). However, single allele gene replacements of MAPK1 yielded viable cells, albeit with reduced MAPK1 abundance. We tested the impact of reduced MAPK1 levels on *L. donovani* gene expression. Figure 3 depicts changes in the expression of heat shock proteins due to modulation of expression of MAPK1 in *L. donovani* promastigotes. Interestingly, reduced expression of MAPK1 in the single allele deletion mutant (Dd8+/−) resulted in significantly decreased expression of the foldosome subunits. Specifically, the expression of HSP90 and HSP70 was decreased by 2–2.5 folds in the single allele deletion mutant (Fig. 3B). The co-chaperones STI1 and SGT could not be detected at all in the lysate from the Dd8+/− mutant (Fig. 3A).

On the other hand, over expression of MAPK1 did not affect the abundance of HSP90 or HSP70 significantly in stably transfected *L. donovani* (Dd8++/++). However, STI1 exhibited increased expression (1.5-fold) in MAPK1 over expressing transfectants (Dd8++/++). The data suggest that LdMAPK1 levels affect the expression of molecular chaperones in *Leishmania*.

### MAPK1 phosphorylates HSP70 and HSP90 *in vitro*

To determine whether MAPK1 can phosphorylate any or both heat shock proteins, an *in vitro* kinase assay was performed using both recombinant proteins as substrate. Myelin basic protein (MBP) was used as reference positive control substrate while bovine serum albumin (BSA) served as a negative control. Autohydrolysis of ATP was also taken into account while measuring the kinase activity. Kinase activity was measured as ATP consumption during incubation with HSPs as substrate. To account for the intrinsic ATPase activity of HSP90 and HSP70^23, 24^, ATP consumption was also measured in the absence of MAPK1. Any ATP consumption in excess was interpreted as resulting from phosphorylation of HSPs by MAPK1.

**Figure 4:**
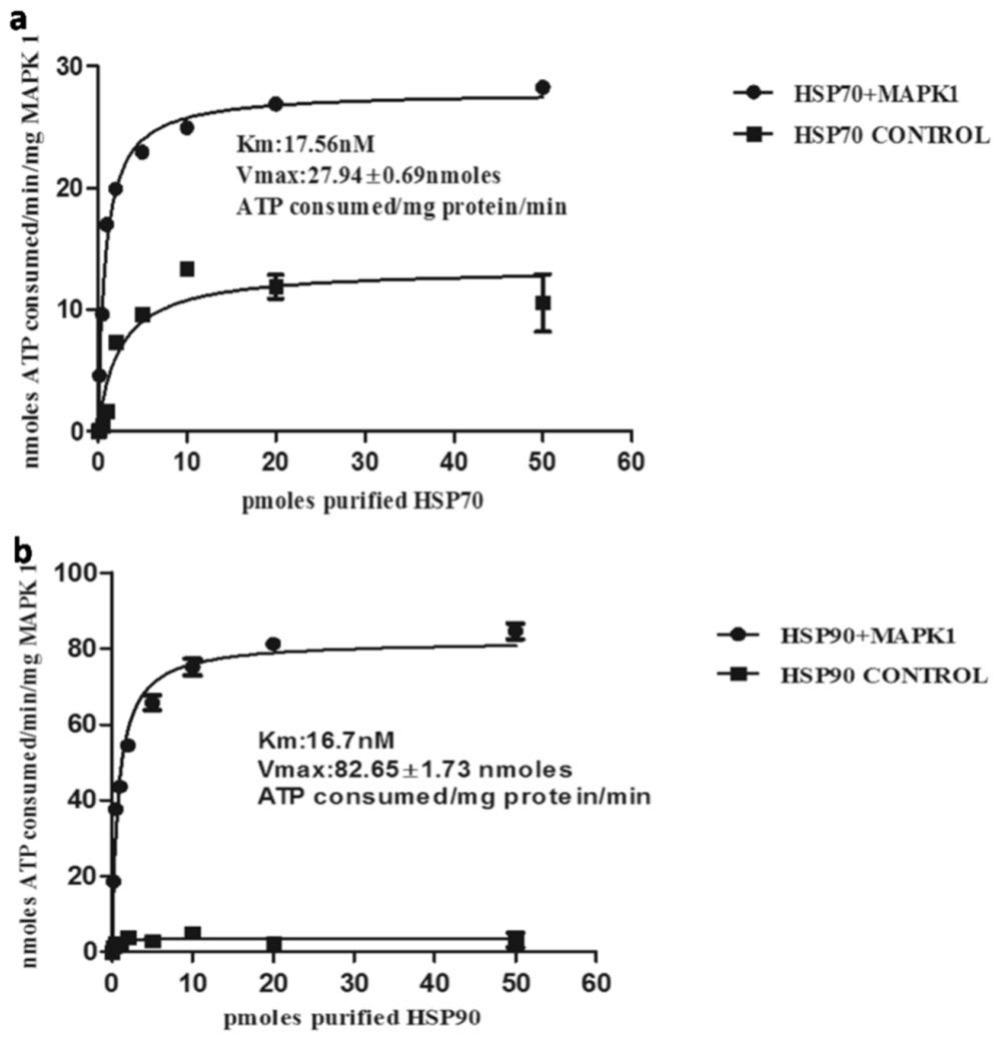
Michaelis Menton plot: LdMAPK1 kinase activity with HSP70 (**a**) and HSP90 (**b**) as substrates. Each data represent mean ± SD from three independent experiments. Inset shows Km and Vmax for the respective protein.

Figure 4 shows that ATP consumption (=kinase activity) increases linearly in the presence of HSP90 or HSP70, in a concentration-dependent manner, and for up to 45 min. The kinase activity follows the Michaelis-Menten equation. The Km for HSP70 and HSP90 was found to be 17.56 nM and 16.70 nM, respectively, with the Vmax at 27.94 ± 0.69 nmoles ATP consumed/mg protein/min and 82.65 ± 1.73 nmoles ATP consumed/mg protein/min.

**Figure 5:**
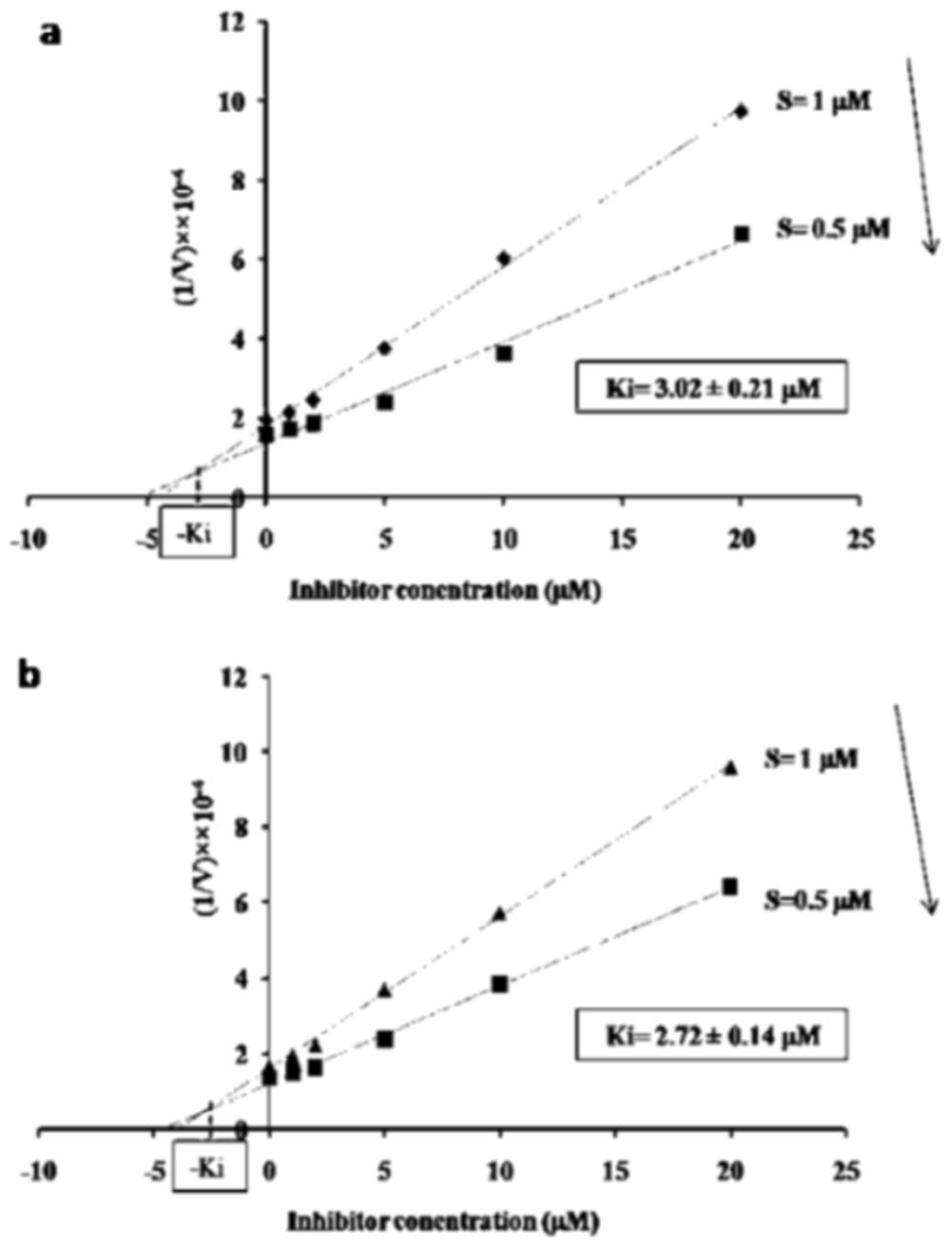
Dixon plot:Competitive inhibition of LdMAPK1 kinase activity by AMTZD with HSP70 (**a**) or HSP90 (**b**) as substrates. The x-coordinate of point of intersection of trend lines represent Ki for the inhibitor.

**Figure 6:**
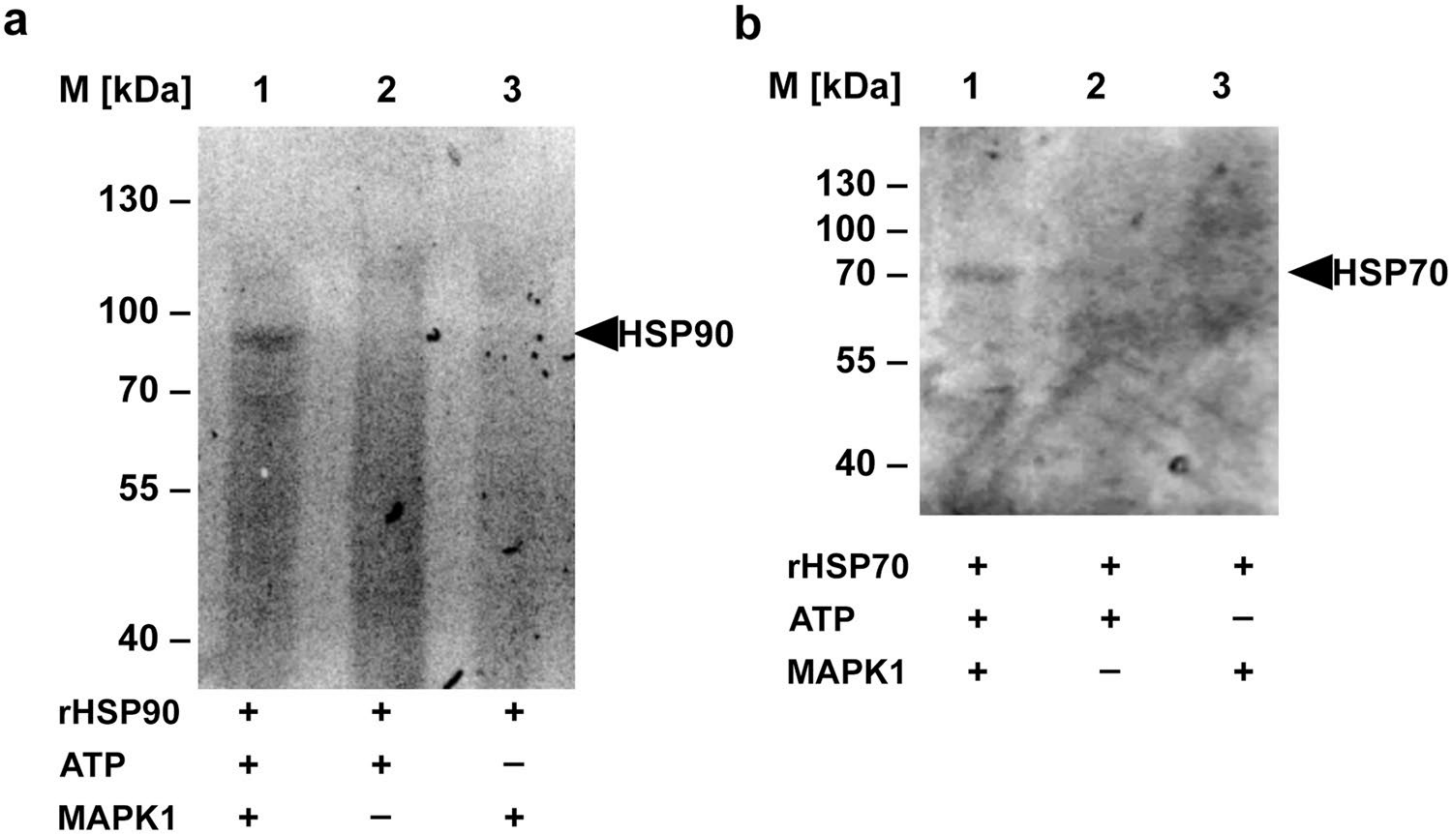
*In vitro* phosphorylation of recombinant HSP70 and HSP90 by LdMAPK1. Relative level of phosphorylation of (**a**) HSP70 (**b**) HSP90 by LdMAPK1 was quantified by ProQ diamond staining (lanes 1–3). Images were analyzed on ChemiDoc XRS+. The molecular masses (kDa) are indicated on the left of (**a**) and (**b**).

**Figure 7:**
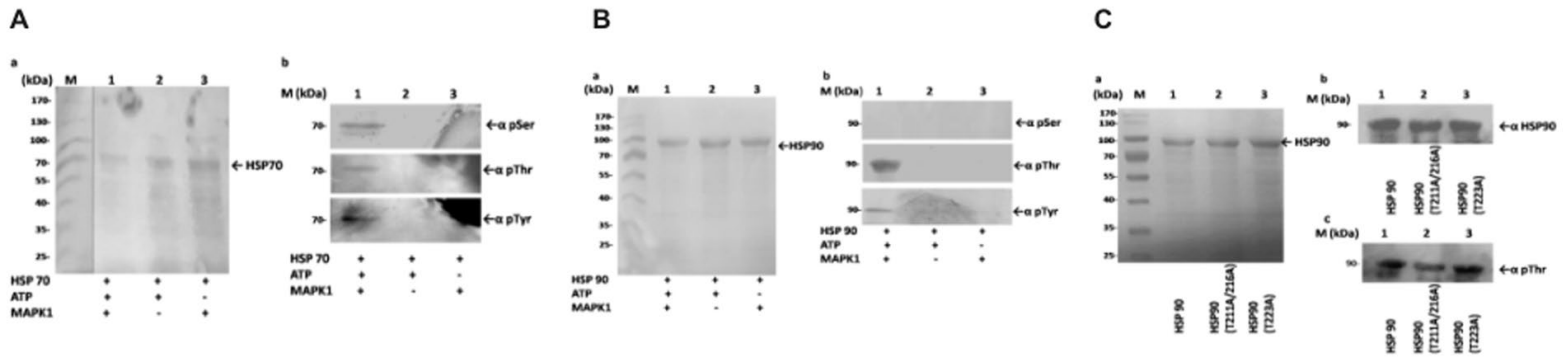
*In vitro* demonstration of LdMAPK1 mediated phosphorylation by western blot analysis. Recombinant HSP70 (**A**{a}) and HSP90 (**B**{a}) and HSP90 site directed mutants (**C**{a}) depicts loading controls. Prior to antibody incubations, Ponceau S staining of membrane for total protein precipitated with TCA after *in vitro* kinase reaction. **A**(b), **B**(b) and **C**(c) depicts western Blot analysis using anti-phosphoserine(pSer)/phosphothreonine(pThr)/phosphotyrosine(pTyr) monoclonal antibody (lanes 1–3). **C**(b) Shows western Blot analysis using anti- HSP90 polyclonal antibodies. The molecular masses (kDa) are shown on the left of (a) and (b).

The *in vitro* MAPK1-mediated kinase activity was inhibited by a known ERK1/2 inhibitor, 3-(2-aminoethyl)-5-([4-ethoxyphenyl] methylene)-2, 4-thiazolidinedione HCl (AMTZD), in a dose-dependent manner (Fig. 5). The Ki for HSP70 and HSP90 phosphorylation was estimated to be 3.02 ± 0.21 µM and 2.72 ± 0.14 µM, respectively. To ascertain that MAPK1 indeed mediates ATP-dependent phosphorylation of HSP70 and HSP90, proteins were precipitated after the kinase reaction, separated by SDS-PAGE, stained with Pro-Q diamond phosphoprotein gel stain (Invitrogen/Molecular probes) and analyzed by Western blotting using anti-phosphoserine, anti-phosphothreonine, and anti-phosphotyrosine monoclonal antibodies. Interestingly, both HSP70 and HSP90 exhibited significant phosphorylation by proQ diamond staining (Fig. 6). HSP70 also showed phosphorylation at serine, threonine and tyrosine residues (Fig. 7A{b}) while HSP90 displayed phosphorylation only at threonine and tyrosine sites, but not on serine residues (Fig. 7B{b}). Interestingly, mutation of three known threonine phosphorylation sites of HSP90 (T211A/216 A or T223A), did not result in a loss of HSP90 phosphorylation by MAPK1 (Fig. 7C{c}).

**Figure 8:**
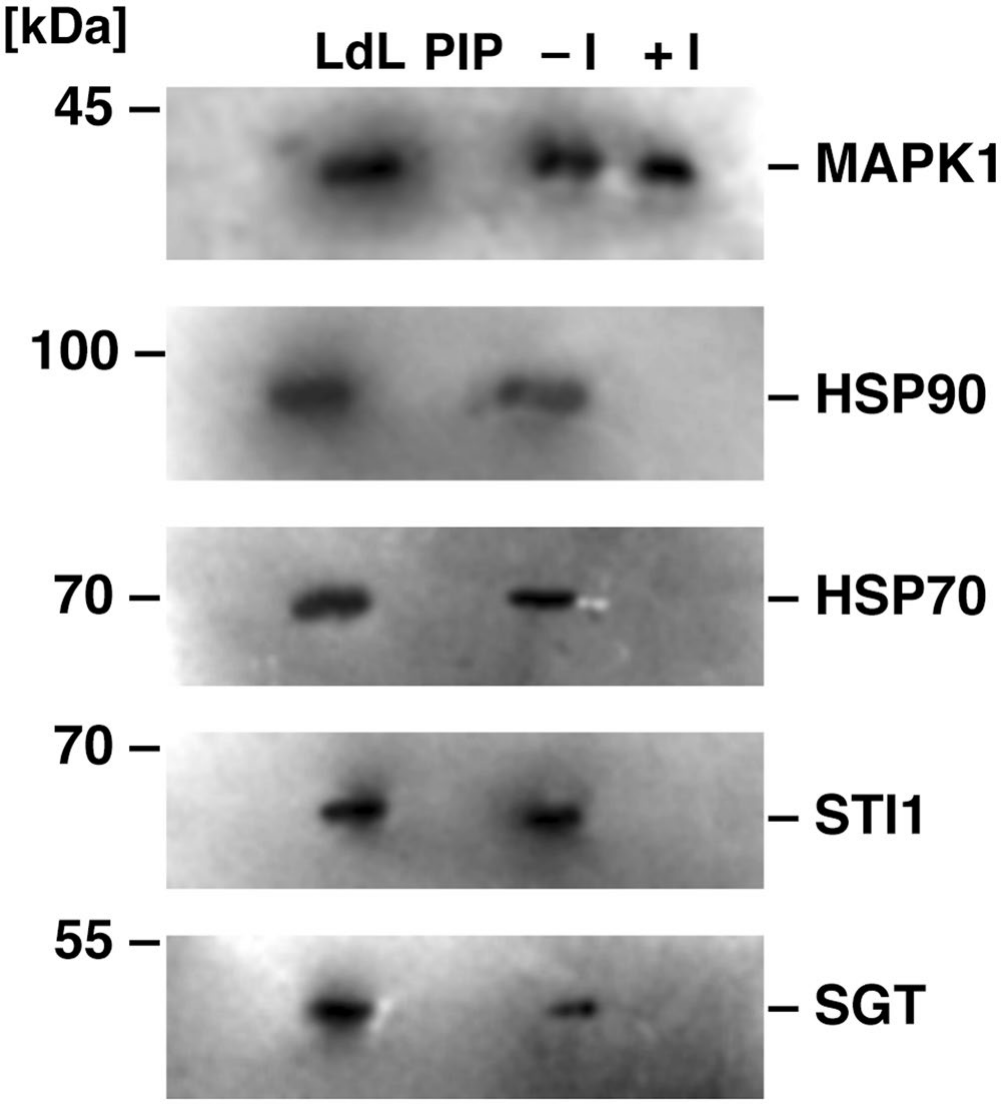
Western blot analysis of co-immunoprecipitation with anti-LdMAPK1 polyclonal antibodies after treatment with AMTZD. LdL: *L. donovani* cell lysate, PIP: co-immunoprecipitate with preimmune sera, CIP(−I): co-immunoprecipitate with anti-LdMAPK1 sera in absence of AMTZD, CIP(+I): Co-immunoprecipitation with anti-LdMAPK1 sera after AMTZD treatment, were separated on SDS PAGE and analyzed by western blotting (lanes 1–4) with anti-LdMAPK1, anti-HSP90, anti-HSP70, anti-STI1 and anti-SGT antibodies. The molecular masses (kDa) are indicated on left.

To further validate that HSP70 and HSP90 are indeed interacting partners or substrate(s) of LdMAPK1, the substrate binding site of LdMAPK1 was blocked in situ by treating the cells with 5 µM AMTZD. The MAPK1 interacting complexes were then co-immunoprecipitated from the lysates of treated cells using anti-LdMAPK1 antibodies. Interestingly, the co-immunoprecipitates from cells treated with inhibitor did not contain HSP70 and HSP90 anymore (Fig. 8). The data confirmed that both HSP70 and HS90 interact with the substrate binding site of the LdMAPK1 enzyme and strongly suggest that HSP70 and HSP90 are substrates for MAPK1 in *L. donovani*. STI1 and SGT also lost their interaction with MAPK1 in presence of AMTZD (Fig. 8, lane 4) which further suggests that both co-chaperones may be interacting with LdMAPK1 indirectly through HSP70 and/or HSP90 as parts of foldosome complex.

## Discussion

MAP kinases (MAPK), the farthest downstream kinases in signal transduction cascades, are highly conserved serine/threonine-specific protein kinases in all eukaryotes. They relay external signals and can ultimately lead to changes in the gene expression profiles. MAPKs regulate critical cellular activities such as cell growth, differentiation, cell shape, motility, cellular stress responses, and apoptosis^25^. They act by phosphorylating their substrates in response to the signal from upstream kinases, MAP kinase kinases.

The life cycle of *Leishmania donovani*, a protozoan parasite and causative agent of fatal visceral leishmaniasis is digenetic reflecting two distinct environments differing in temperature, pH, carbon sources and stress factors. Out of the 15 putative MAPKs identified by genome analysis^13, 26^, only five (MAPK1^14^, MAPK2^20^, MAPK3^16^, MAPK7^12^ and MAPK9^17^) have been functionally characterized by reverse genetics. MAPK1of *L. donovani* has also been shown to play a role in antimony resistance^15^. It negatively regulates the expression of P-glycoprotein-type efflux pumps in the parasite. The decrease in efflux pump activity following an increase in LdMAPK1 expression may cause increased antimony accumulation in the parasite, making it more vulnerable to the drug. Since trans-regulatory transcription factors are notably absent from the *Leishmania* proteome, no information is available about the endpoints of MAP kinase signaling cascades in the parasites. Recently, *L. major* MAPK2 was shown to regulate antimony resistance by phosphorylating an influx pump, AQP1^20^. To identify the possible target proteins of Ld MAPK1, interacting proteins were enriched using anti-MAPK1 antibodies and analysed by SDS-PAGE followed by MS-MS analysis (Fig. 1, Table 1, Supplementary Information: Table S1). Out of 15 proteins, four were identified as subunits of the *Leishmania* HSP90 foldosome complex. Like mammalian HSP90^21^, *Leishmania* HSP90 also has been reported to form a 470kD foldosome complex. This complex likely includes two subunits of HSP90, two subunits of SGT and one subunit each of HSP70, HOP/STI1, and HIP^22, 27^. The ubiquitously expressed HSP90 (also known as HSP83), is the core component of the foldosome complex and plays a pivotal role in promoting the fast growing promastigote stage, and also ensure intracellular amastigote proliferation^28, 29^.

The interaction of MAPK1 with the HSP90 foldosome complex proteins was confirmed by western blot analysis (Fig. 2A). MAPK1 interacted with 4 known subunits of the complex, namely HSP90, HSP70, STI1 and SGT. Under treatment with geldanamycin, MAPK1 interaction with HSP90 and HSP70 persisted, but interaction with STI1 and SGT was lost (Fig. 2B). Geldanamycin is a competitive inhibitor of the HSP90 ATPase activity and inhibits the HSP90 reaction cycle in the foldosome complex, resulting in the destabilization of the complex^30^. Absence of STI1 and SGT from the co-immunoprecipitates after geldanamycin treatment indicates the disruption of the HSP90 foldosome complex. The data suggests that MAPK1 interacts directly with HSP90 and HSP70 and only indirectly with STI1 and SGT.

To test whether MAPK1 also has any role in maintaining the steady state levels of these proteins, we quantified the expression levels of all subunit proteins in three parasite variants (wild type, over expressing and single allele replacement) using western blot analysis. Indeed, MAPK1 expression modulation affects expression of all foldosome subunits (Fig. 3). The decreased expression of MAPK1 in the single allele deletion mutant (Dd8+/−) resulted in a significantly reduced expression of HSP90 and HSP70, and an almost complete loss of the co-chaperones, namely STI1 and SGT in the single allele replacement mutants compared to wild type cells.

The observed dependence of HSP70 and HSP90 expression on MAPK1 abundance is in good agreement with a recent study that showed a regulation of HSP70 expression by MAPKs in myoblast cells^31^. It is also shown that the reduced endogenous expression of HSP90/HSP70 in null mutants of JNK1 affects the protective role of these chaperones in stabilizing HIF-1α^32^. ERK1/2 also induces the expression of HSP40/HSP70, thus protecting mesothelioma cells from heat stress^33^.

MAPK1 may regulate the steady state levels of chaperones and co-chaperones by phosphorylating them. Decreased phosphorylation of HSPs due to decreased levels of MAPK1 may render them less stable and hence, subject to proteolysis. Further, in a phosphoproteome analysis of *L. donovani* promastigotes^9^, three subunits of the foldosome complex, namely HSP90, HSP70 and STI1, were identified as phosphoproteins. To confirm this possibility, phosphorylation of HSP70, HSP90 and STI1 by MAPK1 was tested by an *in vitro* kinase assay. Interestingly, MAPK1 displayed equal kinase activity with HSP70 and HSP90 as substrates (Fig. 4), as the Km for HSP70 and HSP90 was found to be comparable. However, the Vmax for HSP90 was approximately three times higher than that of HSP70. Comparable Km indicates an equal affinity of the enzyme for both substrates while the higher Vmax suggests more phosphorylation sites in HSP90. In contrast to the HSPs, STI1 exhibited almost negligible *in vitro* phosphorylation by MAPK1 in the kinase assay (Vmax; 2.64 nmoles ATP consumed/mg protein/min), suggesting that STI1 may not be a direct substrate of MAPK1. In earlier reports, phosphoproteomic screening demonstrated the phosphorylation of several chaperones including HSP90 and HSP70 in rat mesangial cells by the serine-threonine kinase Akt^34^. HSP90 is a phosphoprotein and its steady-state phosphorylation level is influenced by different cellular environments in a species-specific manner. A recent report has also shown that phosphorylation of yeast HSP90 by the serine-threonine kinase CK2 and by the tyrosine kinase Swe1 respectively, modulates its chaperone function and drug sensitivity^35, 36^. *Leishmania* chaperones and co-chaperones are also highly phosphorylated^37^, and the two HSPs; HSP70 and HSP90, are confirmed substrates of MAPK1 *in vitro* (this paper).

MAPK1-mediated phosphorylation of both HSP70 and HSP90 was further ascertained by ProQ diamond staining (Fig. 6a and b) followed by western analysis (Fig. 7). Both methods confirmed that both HSP90 and HSP70 are phosphorylated by MAPK1 *in vitro*. HSP70 was phosphorylated at serine, threonine and tyrosine sites (Fig. 7A{b}) while HSP90 was phosphorylated only at threonine and tyrosine sites, but not at serine sites (Fig. 7B{b}). Manual analysis of the mass spectrometry spectra predicted phosphorylation sites at HSP90 Thr223 and Ser526^12^. However, the mutation of the two threonine sites did not result in a loss of phosphorylation of HSP90 (Fig. 7C{c}), suggesting the presence of additional target sites for LdMAPK1. In addition to these three sites, eight more putative phosphorylation sites have been identified in *Leishmania* HSP90. Analysis of these sites is ongoing (A.H.-B., unpublished results). Moreover, the analysis was focused on phosphorylation under promastigote-to-amastigote differentiation conditions using MS/MS mass spectrometry^12^. Additional phosphorylation sites may have escaped detection due to the limited coverage of mass spectrometric analysis.

HSP70 phosphorylation has not been studied well in *Leishmania* so far. However, in budding yeast, the phosphorylation of HSP70 by CDK has been reported to regulate cell cycle progression^38^. In *L. infantum* the loss of a different HSP70 family member resulted in a growth arrest at the G2/M phase and a decreased intracellular survival^39^. Moreover, interactions of STI1 and SGT with HSP70 and HSP90 are also essential for survival and proliferation of promastigotes and amastigotes^22, 29^.

The inhibition of MAPK1 activity in the presence of the ERK inhibitor, AMTZD, further validated the enzymatic phosphorylation of HSPs by MAPK1. Since, AMTZD exhibited a competitive type of inhibition (Fig. 5), it should compete with any substrates (HSP70, HSP90 or others) for binding to MAPK1 and might reduce their interaction with the enzyme. On the other hand, AMTZD may or may not hamper the interaction of MAPK1 with its interacting partners. This phenomenon was confirmed by a pull down assay. The treatment of the cells with 5 µM AMTZD resulted in the loss of interaction between MAPK1 and both HSP70 and HSP90 (Fig. 8), suggesting that both HSPs interact with substrate binding site of the LdMAPK1 enzyme and are therefore likely substrates of MAPK1 in *L. donovani*.

Taken together, the present study revealed for the first time a novel role of MAP kinase1 of *L. donovani* in the regulation and post-translational modification of heat shock proteins. It interacts with HSP70 and HSP90 inside the HSP90 foldosome complex. By phosphorylating the two HSPs, MAPK1 may regulate the stability and chaperone function of the foldosome. The signal transduction pathway described herein is also useful for understanding similar pathways in other human-pathogenic Trypanosomatidic parasites.

## Material and Methods

### Materials

Antisera to LdMAPK1 and LdTCP-γ recombinant proteins were raised in rabbit and mice, respectively^40, 41^. Antisera to HSP90, HSP70, STI1 and SGT were raised in chicken^22, 29^. Geldanamycin was purchased from Calbiochem (Merck) and 3-(2-aminoethyl)-5-([4-ethoxyphenyl] methylene)-2, 4-thiazolidinedione HCl (AMTZD) from Sigma-Aldrich. Anti-mice and anti-chicken antibodies were purchased from GE Healthcare and Santa Cruz, respectively. Bacterial strain BL21 (DE3) [pAPlaqIQ] and the pJC45^42^ derivatives pJC45-HSP70, pJC45-HSP90 and pJC45-STI1 were used to express recombinant HSP70, HSP90 and STI1.

### Parasite culture and treatment

*L. donovani* promastigotes (WHO designation MHOM/IN/80/Dd8), originally obtained as a gift from (late) Prof. P.C.C. Garnham and routinely maintained at the Central Drug Research Institute in golden hamsters, were used in the present study. Promastigotes were grown in medium 199 (Sigma) supplemented with 10% (v/v) heat-inactivated FBS (Life Technologies) and 1% (v/v) antibiotic/antimycotic solution (Sigma) as described before^40^. For some studies, parasites were treated with 100 nM Geldanamycin or 2 µM ERK1/2 inhibitor 3-(2-aminoethyl)-5-([4-ethoxyphenyl] methylene)-2, 4-thiazolidinedione HCl (AMTZD) for 24 hours at 24 °C.

LdMAPK1 ORF, cloned into pKS-neo shuttle vector, was transfected into laboratory strain promastigotes (Dd8+/+) as described earlier^15^. Transfectants carrying the LdMAPK1 construct (Dd8++/++) or the vector control (Dd8Vc) were selected and maintained in presence of G418 (40 µg/ml) in M199 medium supplemented with 10% FBS.

Two alleles of LdMAPK1 were targeted for replacement using neomycin and hygromycin phosphotransferase genes flanked by ~1,000 bp of non-coding 5′- and 3′-flanking DNA from the LdMAPK1 locus^43^. Both alleles were replaced with open reading frames of hygromycin and neomycin respectively by homologous recombination. The respective transfectants were selected and maintained under 10 µg ml^−1^ hygromycin and 20 µg ml^−1^ G418 drug pressure in M199 medium supplemented with 20% (v/v) FBS. Since, the growth rate of double allele replacement mutants was severely retarded yielding insufficient cell numbers^43^, all following experiments were carried out using a single allele replacement mutant, Dd8+/−.

### Ethical statement

Institutional Animal Ethics Committee (IAEC) of CSIR-Central Drug Research Institute, Lucknow, reviewed and approved the animal protocols for rabbit, mice and hamsters (IAEC/124/Renew 05(181/14), which was adhered to National guidelines CPCSEA (Committee for the Purpose of Control and Supervision of Experiments and Animals) of Government of India. Animals were housed in plastic/metal cages in climatically controlled rooms and fed with standard rodent food pellet (Lipton India, Bombay) and water and libitum. Rabbit, mice, golden hamsters were not euthanized at any point during study.

Immunisation of laying hens was performed in accordance with §8a, German Animal Protection Law, and registered with the Amt für Gesundheitlichen Verbraucherschutz, Behörde für Umwelt und Gesundheit, Freie und Hansestadt Hamburg.

### Co-immunoprecipitation

Mid log-phase promastigotes (1 × 10^8^) of *L. donovani* were harvested by centrifugation (1,000 × g for 10 min at 4 °C), washed thrice with ice-cold PBS and sonicated in 1 ml lysis buffer (15 mM HEPES pH 7.2, 10 mM MgCl2, 150 mM NaCl, 2 mM EDTA, 5 mM EGTA, 0.5% (v/v) NP-40, 10 mM sodium fluoride, 25 mM sodium orthovanadate and protease inhibitor cocktail{Sigma}). The lysate was centrifuged at 10,000 g for 45 min and a 20 µl aliquot was mixed with sample buffer as ‘cell lysate control’. For the pull down of immune complexes, the remaining supernatant was mixed with 50 µl Dynabeads protein G (Novex, Life Technologies), cross-linked with BS3 to preimmune sera or to rabbit anti-LdMAPK1 sera, and incubated for 3 hours at 4 °C. The beads were collected using a magnetic rack (Life Technologies) and were washed thrice with lysis buffer. The trapped protein complex was eluted from the beads in 1× sample buffer at 37 °C for 30 min. The supernatant after magnetic field precipitation was collected and separated by SDS-PAGE. Gels were stained with Colloidal Coommassie Brilliant Blue (R- 250) stain. Protein bands were excised for MS/MS analysis.

### In gel digestion

Gel slices were rinsed in 25 mM ammonium bicarbonate (ABC) and dehydrated with 25 mM in solution A (acetonitrile/50 mM ABC, 2/1) for 5 min. The gel slices were dried under vacuum for 5–10 min, rehydrated in 25 mM ABC with 100–120 ng trypsin and incubated on ice for 60 min. The gel slices were then incubated at 37^°^C overnight. The peptides were extracted with 50%ACN, 0.1% TFA and the solvent was completely evaporated in a Speed Vac (Savant). The dried tryptic peptides were re-suspended in 30%ACN, 0.1%TFA (5–10 µl) and further processed for MALDI MS/MS analysis.

### Western blotting

Production of SDS cell lysates, discontinuous SDS-PAGE and Western blotting were performed according to standard protocols^44, 45^. The proteins from immunoprecipitation experiments (1 × 10^8^ cells) and cell lysate (2 × 10^6^ parasites) of wild type cells, over-expressing and single allele deletion mutants were separated by SDS-PAGE (10%) and transferred onto nitrocellulose membranes. The membranes were treated with blocking solution (3% BSA and 0.1% Tween-20 in phosphate-buffered saline), before they were probed using monoclonal (anti-phosphoserine/phosphothreonine/phosphotyrosine), or polyclonal primary antibodies (anti-MAPK1, anti-HSP90, anti-HSP70, anti-STI1, anti-SGT) (1:3000 in blocking solution), or followed by incubation with an anti-rabbit or anti-chicken mouse-IgG-HRP conjugate (1:10,000 in phosphate buffered saline with 0.1% Tween-20) as secondary antibody. Blots were developed using ECL reagent and visualized on Hyper film ECL (GE Healthcare). The images were scanned and a quantitative assessment was carried out with software provided within the Gel-Doc System (Alpha Innotech).

### Recombinant Expression of MAPK1, HSP90, HSP70 and STI1

Recombinant LdMAPK1 protein (rLdMAPK1) was expressed and purified as described before^15^. Briefly, the expression construct, pGEX-KG-LdMAPK1, was transformed in BL21 (DE3)-Plys cells to express GST fused rLdMAPK1. The expression of protein was induced with 0.5 mM IPTG at 24 °C. The expressed protein was purified to homogeneity using Glutathione Sepharose B column (GE Healthcare) according to the manufacturer’s protocol.

The constructs for expression of recombinant HSP70, HSP90 and STI1 proteins, pJC45-HSP70, pJC45-HSP90 and pJC45-STI1, respectively, were introduced into *E. coli* BL21(DE3) [pAPlacI^Q^] and expression was induced using 0.4 mM IPTG at 37 °C for 2 hours. The His-tagged proteins were purified^46^ using Ni-NTA column (Qiagen), as per manufacturer’s instructions. Purity of the proteins was verified by SDS-PAGE and Coomassie Brilliant Blue staining.

### Kinase assay

Kinase assay of purified native recombinant protein rLdMAPK1 was performed with myelin basic protein (MBP; Sigma) as standard kinase substrate and ATP as phosphate donor. Purified proteins; recombinant HSP70, HSP90, STI1 and HSP90 threonine mutant proteins (T211A/216 A, T223A) were tested as substrates for MAPK1. Single or double amino acid exchanges were introduced by mutagenesis PCR as described^29^.

The kinase activity was analyzed using the ATP utilization method and Kinase-Glo plus luminescent assay kit (Promega), as described earlier^15, 19^. Briefly, a reaction mixture with a total volume of 50 μl containing 50 mM morpholinepropanesulfonic acid (MOPS; pH 7.2), 100 mM NaCl, 10 mM MgCl2, 2 mM MnCl2, 100 μM ATP, 100ng of recombinant MAPK1 and varying concentration of test substrates was incubated at 30 °C for 30 min, and residual ATP was measured using the Kinase-Glo Plus luminescent assay kit (Promega) using a luminometer (Polar Star Galaxy). 10 µg of myelin basic protein (MBP; Sigma) or BSA were used as positive and negative control substrates, respectively. Auto hydrolysis of ATP and intrinsic ATPase activity of HSP90 and HSP70 were monitored in replica assays without MAPK1 enzyme and/or HSP90/HSP70. The assay was standardized using different concentrations of test substrates, HSP70 and HSP90 (0.2–50 pmoles) and STI1 (20–120 pmoles). Km and Vmax were calculated with nonlinear regression using GraphPad Prism software. The kinase inhibition assay was performed using ERK inhibitor 3-(2-aminoethyl)-5-([4-ethoxyphenyl] methylene) - 2, 4-thiazolidinedione HCl (AMTZD) and the Ki was calculated.

Phosphorylation of HSP70 and HSP90 by LdMAPK1 was also confirmed by western analysis using primary monoclonal (anti-phosphoserine, anti-phosphothreonine and anti-phosphotyrosine) antibodies. Firstly, the kinase reaction was performed using HSP70, HSP90 and site directed HSP90 mutant protein(s) as substrates, LdMAPK1 as enzyme and ATP as phosphate donor. After one hour of incubation, the proteins were precipitated with trichloroacetic acid, separated by SDS PAGE, blotted on nylon membrane, decorated with anti-phosphothreonine (pThr)/-phosphotyrosine(pTyr)/-phosphoserine (pSer) monoclonal antibodies (1:1000, Cell signalling Technology, USA) and visualized using anti-mouse/HRP conjugates.

## Electronic supplementary material

Table S1

## References

[1] Alvar J (2008). The relationship between leishmaniasis and AIDS: the second 10 years. Clin Microbiol Rev..

[2] Kedzierski L (2009). Leishmaniasis: current treatment and prospects for new drugs and vaccines. Curr Med Chem..

[3] Machado-Silva A, Guimarães PP, Tavares CA, Sinisterra RD (2015). New perspectives for leishmaniasis chemotherapy over current anti-leishmanial drugs: a patent landscape. Expert Opin Ther Pat..

[4] Mohapatra S (2014). Drug resistance in leishmaniasis: Newer developments. Trop Parasitol..

[5] Diro E (2015). Atypical manifestations of visceral leishmaniasis in patients with HIV in north Ethiopia: a gap in guidelines for the management of opportunistic infections in resource poor settings. Lancet Infect Dis..

[6] Handman E (1999). Cell biology of *Leishmania*. Adv Parasitol..

[7] Rosenzweig D (2008). Retooling *Leishmania* metabolism: from sand fly gut to human macrophage. FASEB J..

[8] Raggiaschi R, Gotta S, Terstappen GC (2005). Phosphoproteome analysis. Biosci. Rep..

[9] Morales MA (2008). Phosphoproteomic analysis of *Leishmania donovani* pro- and amastigote stages. Proteomics..

[10] Tsigankov P, Gherardini PF, Helmer-Citterich M, Späth GF, Zilberstein D (2013). Phosphoproteomic analysis of differentiating *Leishmania* parasites reveals a unique stage-specific phosphorylation motif. J Proteome Res..

[11] Tsigankov P (2014). Regulation dynamics of *Leishmania* differentiation: deconvoluting signals and identifying phosphorylation trends. Mol. Cell Proteomics..

[12] Morales MA (2010). Phosphoproteome dynamics reveal heat-shock protein complexes specific to the *Leishmania donovan*i infectious stage. Proc Natl Acad Sci USA.

[13] Parsons M, Worthey EA, Ward PN, Mottra JC (2005). Comparative analysis of the kinomes of three pathogenic trypanosomatids: *Leishmania major, Trypanosoma brucei* and *Trypanosoma cruzi*. BMC Genomics..

[14] Wiese M (1998). A mitogen-activated protein (MAP) kinase homologue of *Leishmania mexicana* is essential for parasite survival in the infected host. EMBO J..

[15] Ashutosh (2012). Downregulation of mitogen-activated protein kinase 1 of *Leishmania donovani* field isolates is associated with antimony resistance. Antimicrob Agents Chemother..

[16] Erdmann M, Scholz A, Melzer IM, Schmetz C, Wiese M (2006). Interacting protein kinases involved in the regulation of flagellar length. Mol Biol Cell..

[17] Bengs F, Scholz A, Kuhn D, Wiese M (2005). LmxMPK9, a mitogen-activated protein kinase homologue affects flagellar length in *Leishmania mexicana*. Mol Microbiol..

[18] Kuhn D, Wiese M (2005). LmxPK4, a mitogen-activated protein kinase kinase homologue of *Leishmania mexicana* with a potential role in parasite differentiation. Mol Microbiol..

[19] John von Freyend S (2010). LmxMPK4, an essential mitogen-activated protein kinase of *Leishmania mexicana* is phosphorylated and activated by the STE7-like protein kinase LmxMKK5. Int J Parasitol..

[20] Mandal G (2012). Modulation of *Leishmania major* aquaglyceroporin activity by a mitogen-activated protein kinase. Mol Microbiol..

[21] Buchanan G (2007). Control of androgen receptor signalling in prostate cancer by the co-chaperone small glutamine rich tetracopeptide repeat containing protein alpha. Cancer Res..

[22] Ommen G, Chrobak M, Clos J (2010). The co-chaperone SGT of *Leishmania donovani* is essential for the parasite’s viability. Cell Stress Chaperones..

[23] Flaherty KM, Wilbanks SM, DeLuca-Flaherty C, McKay DB (1994). Structural basis of the 70-kilodalton heat shock cognate protein ATP hydrolytic activity. II. Structure of the active site with ADP or ATP bound to wild type and mutant ATPase fragment. J Biol Chem..

[24] Jackson SE (2013). Hsp90: Structure and function. Top Curr Chem..

[25] Chen Z (2001). MAP kinases. Chem Rev..

[26] Ivens AC (2005). Leishmaniasis: current treatment and prospects for new drugs and vaccines. Curr Med Chem..

[27] Webb JR, Campos-Neto A, Skeiky YA, Reed SG (1997). Molecular characterization of the heat-inducible LmSTI1 protein of *Leishmania major* [In Process Citation]. Mol Biochem Parasitol..

[28] Wiesgigl M, Clos J (2001). The heat shock protein 90 of *Leishmania donovani*. Med Microbiol Immunol..

[29] Hombach A, Ommen G, Chrobak M, Clos J (2013). The Hsp90-Sti1 interaction is critical for *Leishmania donovani* proliferation in both life cycle stages. Cell Microbiol..

[30] Neckers L (2003). Development of small molecule Hsp90 inhibitors: utilizing both forward and reverse chemical genomics for drug identification. Curr Med Chem..

[31] Bironaite D, Brunk U, Venalis A (2013). Protective induction of Hsp70 in heat-stressed primary myoblasts: Involvement of MAPKs. J Cell Biochem..

[32] Zhang D, Li J, Costa M, Gao J, Huang C (2010). JNK1 mediates degradation HIF-1alpha by a VHL-independent mechanism that involves the chaperones Hsp90/Hsp70. Cancer Res..

[33] Roth M, Zhong J, Tamm M, Szilard J (2009). Mesothelioma cells escape heat stress by upregulating Hsp40/Hsp70 expression via mitogen-activated protein kinases. J Biomed Biotechnol..

[34] Barati MT, Rane MJ, Klein JB, McLeish KR (2006). A proteomic screen identified stress-induced chaperone proteins as targets of Akt phosphorylation in mesangial cells. J Proteome Res..

[35] Mollapour M (2010). Swe1Wee1-dependent tyrosine phosphorylation of Hsp90 regulates distinct facets of chaperone function. Mol Cell..

[36] Mollapour M, Tsutsumi S, Kim YS, Trepel J, Neckers L (2011). Casein kinase 2 phosphorylation of Hsp90 threonine 22 modulates chaperone function and drug sensitivity. Oncotarget..

[37] Hombach A, Clos J (2014). No stress–Hsp90 and signal transduction in *Leishmania*. Parasitology..

[38] Truman AW (2012). CDK-dependent Hsp70 Phos.phorylation controls G1 cyclin abundance and cell-cycle progression. Cell..

[39] Folgueira C (2008). Effects of the disruption of the HSP70-II gene on the growth, morphology, and virulence of *Leishmania infantum* promastigotes. Int Microbiol..

[40] Ashutosh GS, Ramesh Sundar S, Goyal N (2005). Use of *Leishmania donovani* field isolates expressing the luciferase reporter gene in in vitro drug screening. Antimicrob Agents Chemother..

[41] Bhaskar KN, Goyal N (2012). Cloning, characterization and sub-cellular localization of gamma subunit of T-complex protein-1 (chaperonin) from *Leishmania donovani*. Biochem Biophys Res Com..

[42] Schlüter A (2010). Expression and subcellular localization of cpn60 protein family members in *Leishmania donovani*. Biochem Biophys Acta..

[43] Garg M, Goyal N (2015). MAPK1 of *Leishmania donovani* modulates antimony susceptibility by down regulating P-glycoprotein efflux pumps. Antimicrob Agents Chemother..

[44] Laemmli UK (1970). Cleavage of structural proteins during the assembly of the head of bacteriophage T4. Nature..

[45] Towbin H, Staehelin T, Falgout B (1979). Electrophoretic transfer of proteins from polyacrylamide gels to nitrocellulose sheets: procedure and some application. Proc Natl Acad Sci USA..

[46] Clos J, Brandau S (1994). pJC20 and pJC40: two high-copy-number vectors for T7 RNA polymerasedependent expression of recombinant genes in Escherichia coli. Protein Express. Purif..

